# Successful treatment of recalcitrant cutaneous lupus erythematosus with anifrolumab in patients without systemic lupus erythematosus

**DOI:** 10.1016/j.jdcr.2024.11.014

**Published:** 2024-11-24

**Authors:** Bishoy Abdelmalik, Steven Svoboda, Pooja Gurnani, Kiran Motaparthi

**Affiliations:** a University of Florida College of Medicine, Gainesville, Florida; b Department of Dermatology, University of Florida College of Medicine, Gainesville, Florida

**Keywords:** anifrolumab, lupus, recalcitrant, refractory

## Introduction

Lupus erythematosus (LE) is a chronic, multisystem autoimmune disorder which presents with varying phenotypic findings depending on organ involvement. LE subgroups include neonatal lupus erythematosus, drug-induced lupus, systemic lupus erythematosus (SLE), and cutaneous lupus erythematosus (CLE).^1^^,^^2^ CLE may occur in association with or independent of SLE. CLE is further classified into 3 LE-specific cutaneous subtypes based on unique clinical features, histopathology, and immunologic characteristics. These subtypes are acute cutaneous lupus erythematosus, subacute cutaneous lupus erythematosus, and chronic cutaneous lupus erythematosus.^3^

Although CLE is the second most common manifestation of LE, there are no medications which have been Food and Drug Administration-approved for the treatment of CLE.^4^ Management incorporates preventative measures with localized and systemic pharmacotherapy. Prevention focuses on smoking cessation and photoprotection. Current first-line pharmacologic interventions for CLE include topical corticosteroids and antimalarials. Management with alternative nonspecific immunosuppressants is frequently initiated due to treatment failure. Refractory CLE becomes difficult to manage with a limited understanding of treatment progression.^5^^,^^6^

Anifrolumab, a monoclonal antibody against type I interferon receptor recently approved by the Food and Drug Administration for SLE in 2021, reduces the severity of skin disease in patients with SLE and CLE.^7^ Herein, we present 3 distinct subtypes of refractory CLE, without associated SLE, successfully treated with anifrolumab.

## Case presentation

### Patient 1

**Fig 1:**
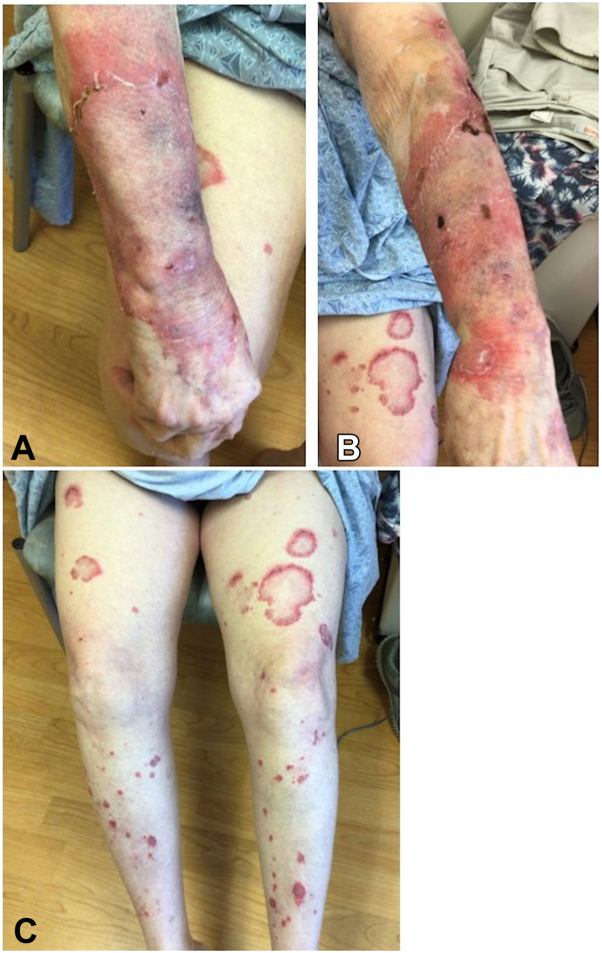
Patient 1 before treatment with anifrolumab. **A** and **B,** Forearms with annular erythematous plaques with surrounding scale. **C,** Anterior thighs and shins with scattered, annular erythematous papules, and plaques with surrounding scale.

A 70-year-old woman with a history of chronic kidney disease and hypothyroidism presented with polycyclic, pink, scaly plaques on all extremities and trunk in a predominantly photo-distributed pattern (Fig 1). Laboratory evaluation revealed positive antinuclear antibodies (ANA) with a 1:320 titer in a speckled pattern, positive Sjogren antibody, and negative anti-Smith and double-stranded DNA antibodies. A punch biopsy showed an atrophic epidermis with a vacuolar interface dermatitis, significant mucin, and a perivascular and periadnexal lymphocytic infiltrate, confirming the clinical diagnosis of subacute cutaneous lupus erythematosus. According to the European League Against Rheumatism and American College of Rheumatology criteria, this patient did not meet criteria for SLE based on a score of 4. Furthermore, a review of medications of medications and the onset of cutaneous findings permitted exclusion of medication-induced CLE.

**Fig 2:**
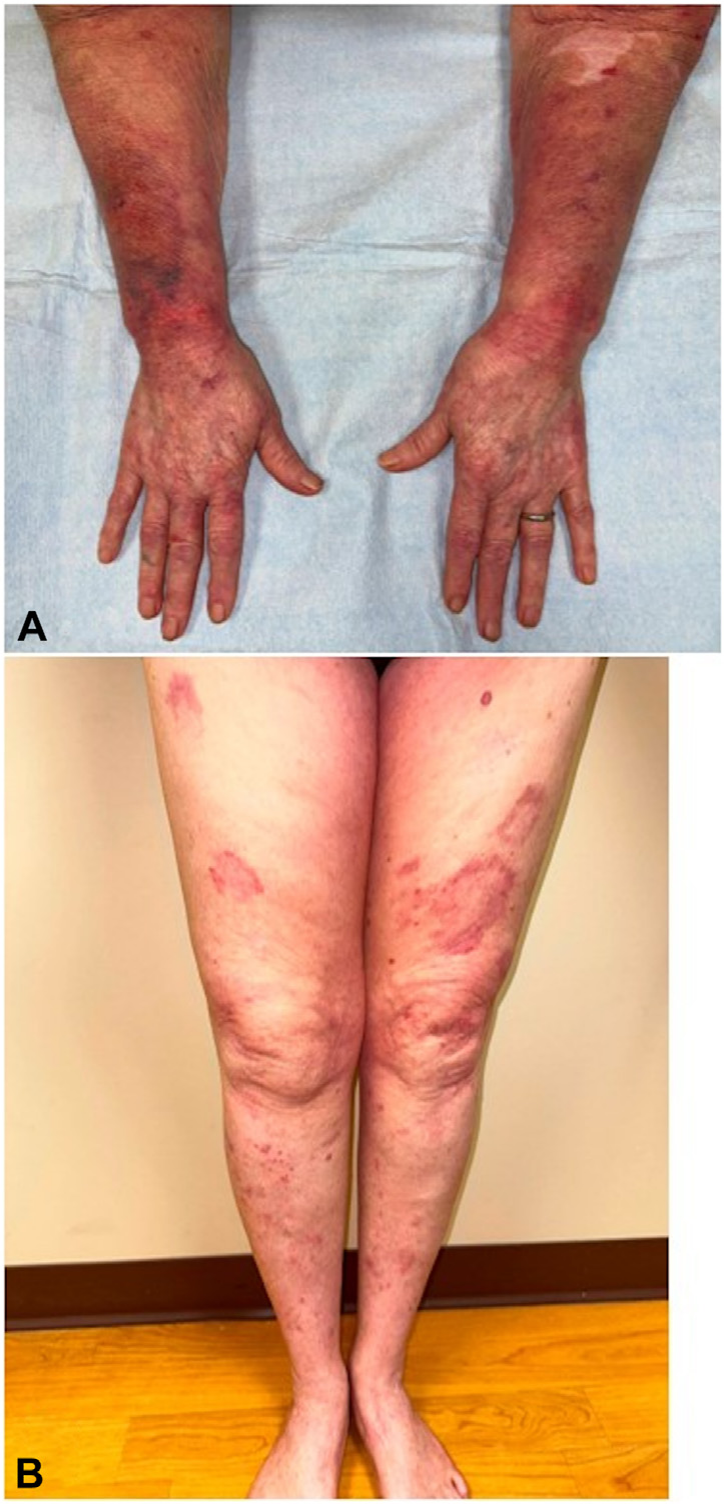
Patient 1 at follow-up after 2 infusions of anifrolumab (**A**) Forearms with postinflammatory erythema. **B,** Anterior thighs and shins with improving resolution of scale and deep erythema.

Prior treatments, including topical and systemic corticosteroids, hydroxychloroquine, mycophenolate, methotrexate, thalidomide, and intravenous immunoglobulin were attempted, but ultimately discontinued due to factors such as cost, adverse effects, inaccessibility, or lack of efficacy. Hydroxychloroquine, mycophenolate, thalidomide, and methotrexate were trialed for approximately 20 years, 6 years, 2 years, and 2 months, respectively. Due to the refractory nature of her disease, anifrolumab was initiated at 300 mg every 4 weeks. After the first 2 monthly infusions of anifrolumab, the patient reported significant improvement in lesion appearance and associated pruritus. Physical examination showed postinflammatory erythema (Fig 2).

### Patient 2

**Fig 3:**
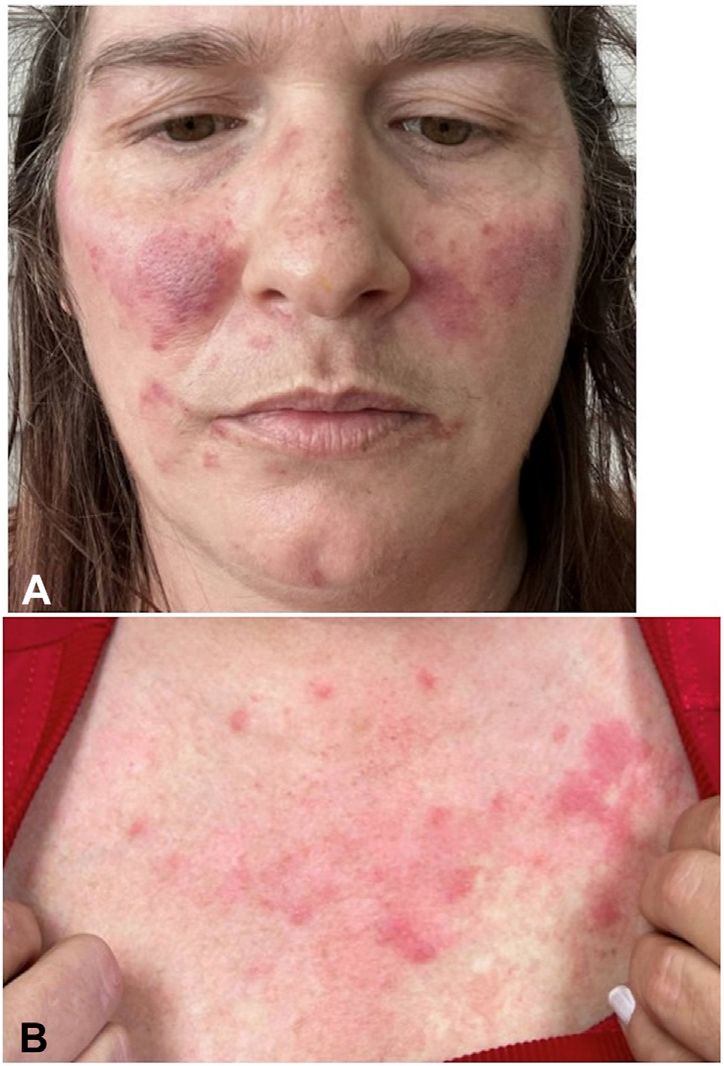
Patient 2 before treatment with anifrolumab. **A,** Erythematous papules and plaques. **B,** Upper chest with grouped erythematous papules and nodules.

A 48-year-old woman presented with a longstanding history of nontender erythematous dermal plaques on the cheeks, forehead, and upper chest (Fig 3). Laboratory evaluation demonstrated positive ANA, with a 1:40 titer, excluding SLE. A punch biopsy revealed superficial and deep perivascular, perifollicular, and perieccrine lymphocytic inflammatory infiltrate with abundant mucin and no interface dermatitis, confirming the clinical diagnosis of tumid lupus erythematosus. A review of the patient’s medications and the onset of cutaneous findings excluded drug-induced CLE.

**Fig 4:**
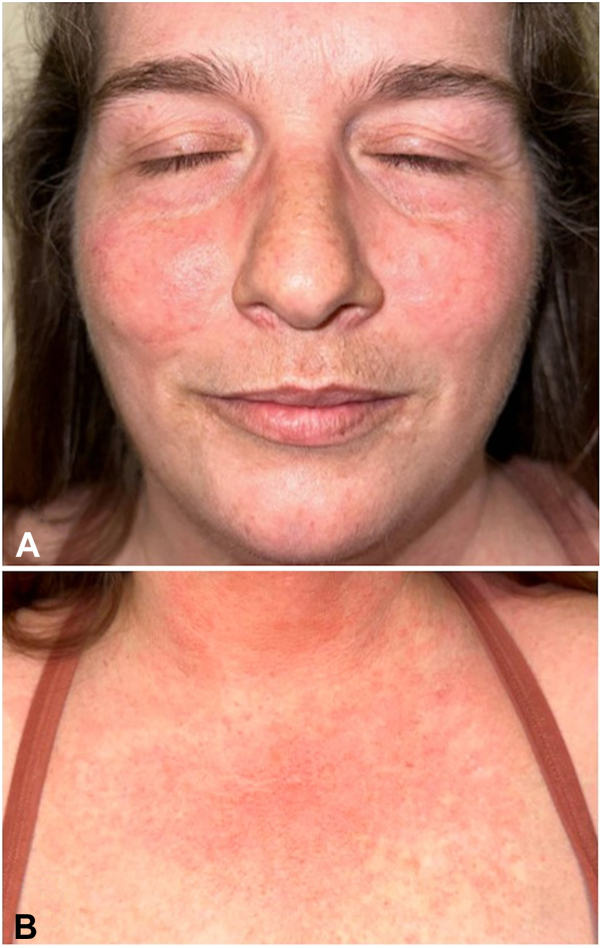
Patient 2 at after 2 infusions of anifrolumab. **A,** Face with resolution of papules and plaques. **B,** Upper chest resolution of papules and plaques and mild postinflammatory erythema.

Photoprotection, topical corticosteroids, hydroxychloroquine, methotrexate, and mycophenolate were ineffective. Hydroxychloroquine, methotrexate, and mycophenolate were trialed for 6 months, 2 months, and 3 months, respectively. After 2 monthly infusions of anifrolumab 300 mg, the patient experienced complete resolution (Fig 4) without new flares.

### Patient 3

**Fig 5:**
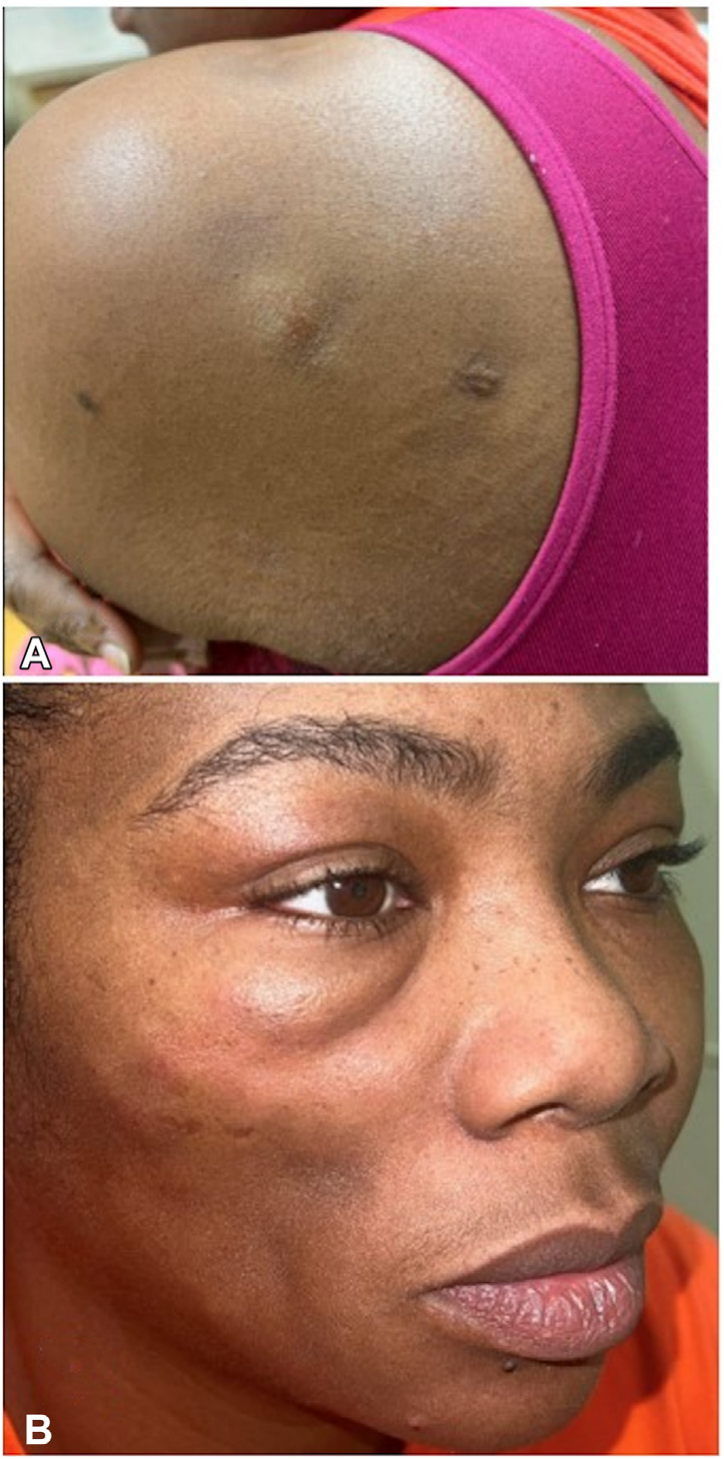
Patient 3 before treatment with anifrolumab. **A,** Posterior shoulder with nodules and adjacent hyperpigmented scars at prior biopsy sites. **B,***Lower* cheek with atrophy and *upper* cheek with nodules.

**Fig 6:**
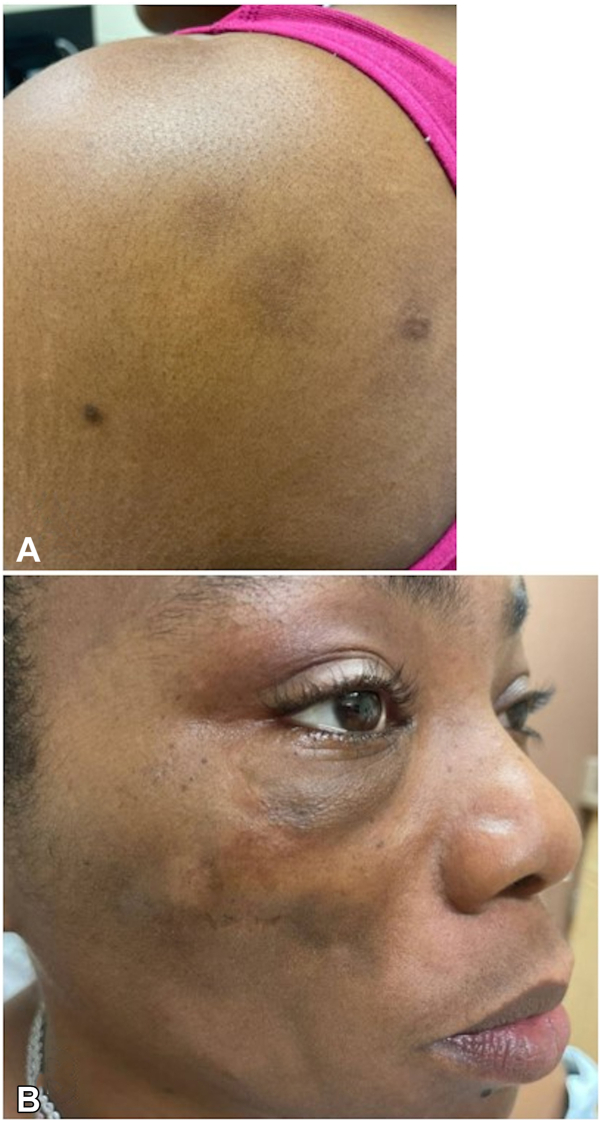
Patient 3 after 3 infusions of anifrolumab. **A,** Posterior shoulder with postinflammatory hyperpigmentation and resolution of nodules. **B,***Lower* cheek with atrophy and *upper* cheek with atrophy, postinflammatory hyperpigmentation, and resolution of nodules.

A 41-year-old woman presented with nodules and atrophic plaques on the shoulder and cheek (Fig 5). A diagnosis of lupus panniculitis was made based on clinical findings as well as typical histopathologic features demonstrating a lymphocytic lobular panniculitis with hyaline necrosis. Laboratory evaluation demonstrated a positive ANA, with an unknown titer. Independent of the ANA titer, the patient did not meet the European League Against Rheumatism and American College of Rheumatology criteria for SLE. A review of the patient’s medications and the onset of cutaneous findings excluded drug-induced CLE. Prior treatments included pimecrolimus, triamcinolone, systemic steroids, methotrexate, hydroxychloroquine, and mycophenolate. Methotrexate and hydroxychloroquine were attempted for multiple months and 2 years respectively until discontinued, while mycophenolate has been ongoing now for 2 years. Following 3 monthly infusions of anifrolumab 300 mg, the patient demonstrated significant improvement in nodules, with follow-up examination demonstrating postinflammatory pigmentary change and atrophy (Fig 6). The patient is currently controlled on a combination of anifrolumab 300 mg monthly and mycophenolate 500 mg twice daily.

## Discussion

CLE is characterized by cytotoxic lesional inflammation resulting from a complex inflammatory cascade driven by increased expression of type I interferons. Targeted therapeutics against this IFN-driven cascade, such as anifrolumab, have been shown to be viable treatment options in reducing the burden of refractory cutaneous disease associated with SLE.^7^, ^8^, ^9^ This series adds to growing number of descriptions documenting the efficacy of anifrolumab in treating recalcitrant CLE. Marked and rapid improvement with anifrolumab was demonstrated in each patient who presented with a different subtype of CLE, without underlying SLE. This series also contains the second reported case of TLE treated successfully with anifrolumab.^10^ The favorable treatment outcomes with anifrolumab in patient without underlying SLE suggests its potential utility for patients with skin-limited disease and underscores the need for robust studies across the various subtypes of CLE to establish a treatment protocol.

No adverse events (AE) related to anifrolumab were observed in these 3 patients. This is consistent with the low rate of AEs documented in the phase III trial of anifrolumab in active SLE (TULIP-2) which included 362 patients. Of AEs reported, upper respiratory tract infection (21.7%), nasopharyngitis (15.6%), infusion-related reactions (13.9%), bronchitis (12.2%), and herpes zoster infection (7.2%) occurred most frequently over the initial 52-week trial period. Serious adverse events including death, a major cardiovascular event, a hypersensitivity reaction, and non-opportunistic serious infections occurred in 8.3% and 17.0% of patients who received anifrolumab versus placebo, respectively.^11^ A placebo-controlled, 3-year long-term extension (LTE) study found that exposure-adjusted incidence rates of AEs and serious adverse events were similar between anifrolumab and placebo groups. Rates of adverse events decreased over time in the LTE study, demonstrating that treatment with anifrolumab was well tolerated with an acceptable long-term safety profile.^12^ Additional data from the TULIP-2 and LTE studies found that anifrolumab demonstrated efficacy in reducing the cutaneous disease burden in patients with SLE for a total of up to 39 monthly doses. Duration of treatment for CLE without SLE remains unclear.^11^^,^^12^ As a result, treatment length is currently dictated by insurance coverage and clinical response.

Similar to other descriptive studies on anifrolumab for CLE, limitations of this series include a small sample size and lack of a comparative group. These patients from a single institution may not be representative of patients with CLE in the general population, limiting external validity. Future studies are needed to expand upon the long-term efficacy, duration of treatment, and durability of response with anifrolumab for CLE without SLE.^7^^,^^8^^,^^13^

## Conclusion

Anifrolumab represents a promising therapeutic option for patients with recalcitrant CLE, even without underlying SLE.

## Conflicts of interest

None disclosed.
